# A combination of process of care and clinical target among type 2 diabetes mellitus patients in general medical clinics and specialist diabetes clinics at hospital levels

**DOI:** 10.1186/s12913-017-2486-7

**Published:** 2017-08-07

**Authors:** Sokha Sieng, Cameron Hurst

**Affiliations:** 1 Faculty of Public Health, Khon Kaen University, Khon Kaen, 40002 Thailand; 2 School Health Department, Ministry of Education, Youth and Sport, Phnom Penh, Cambodia; 3 Office of Dean for Undergraduate Studies Division, University of Cambodia, Phnom Penh, Cambodia; 4 Faculty of Medicine, Chulalongkorn University, Bangkok, 10330 Thailand

**Keywords:** Quality of care, Type 2 diabetes mellitus, General medical clinics, Specialist diabetes clinics, Hospital levels

## Abstract

**Background:**

This study compares a combination of processes of care and clinical targets among patients with type 2 diabetes mellitus (T2DM) between specialist diabetes clinics (SDCs) and general medical clinics (GMCs), and how differences between these two types of clinics differ with hospital type (community, provincial and regional).

**Methods:**

Type 2 diabetes mellitus patient medical records were collected from 595 hospitals (499 community, 70 provincial, 26 regional) in Thailand between April 1 to June 30, 2012 resulting in a cross-sectional sample of 26,860 patients. Generalized linear mixed modeling was conducted to examine associations between clinic type and quality of care. The outcome variables of interest were split into clinical targets and process of care. A subsequent subgroup analysis was conducted to examine if the nature of clinical target and process of care differences between GMCs and SDCs varied with hospital type (regional, provincial, community).

**Results:**

Regardless of the types of hospitals (regional, provincial, or community) patients attending SDCs were considerably more likely to have eye and foot exam. In terms of larger hospitals (regional and provincial) patients attending SDCs were more likely to achieve HbA1c exam, *All FACE* exam, BP target, and the *Num7Q*. Interestingly, SDCs performed better than GMCs at only provincial hospitals for LDL-C target and the *All7Q*. Finally, patients with T2DM who attended community hospital-GMCs had a better chance of achieving the blood pressure target than patients who attended community hospital-SDCs.

**Conclusions:**

Specialized diabetes clinics outperform general medical clinics for both regional and provincial hospitals for all quality of care indicators and the number of quality of care indicators achieved was never lower. However, this better performance of SDC was not observed in community hospital. Indeed, GMCs outperformed SDCs for some quality of care indicators in the community level setting.

## Background

Diabetes Mellitus (DM) is a chronic disease and both the number of people with diabetes and its associated health care costs are projected to increase considerably in both developing and developed countries alike [1–3]. For instance, the prevalence of diabetes in Thailand was 7.7% and spending was 0.5 billion USD in 2010, and these are predicted to rise to 9.8% and 0.7 billion USD by 2030, respectively [1–3]. With the ongoing epidemic of type 2 diabetes, the demand for quality of diabetes care and the need for providing adequate and consistent levels of care require a deeper understanding of the efficacy of existing patients care models. For example, general medical clinic may provide care equivalent to or better than that of specialist diabetes clinic, both in achieving the desired process of care and clinical target, while at the same time, decreasing costs [4, 5].

Achieving the process of care and clinical targets is important because they have been shown to reduce and/or delay the onset of microvascular or macrovascular complications [6–8]. Although, several studies have consistently demonstrated that the better adherence to processes of diabetes care measures does not always lead to better clinical outcomes [9]. However, limited attention has been given to the potential benefits of a combination of processes of care and clinical targets into a composite measure of optimal diabetes care. One recent study demonstrated that specialist diabetes clinics are more likely to be associated with achieving the quality of diabetes care [10]. However, it is less clear how to best integrate general medical clinics into a new health service model. Another important question that needs investigating is what is the effect of hospital type on achieving processes of care and clinical targets, and how might hospital type modify the relative efficacy of general medical clinics. The aim of this study is to evaluate the combination of processes of care and clinical targets among patient with type 2 diabetes mellitus attending either general medical clinics or specialist diabetes clinics for different hospital levels (regional, provincial and community).

## Methods

In this study a proportional to size stratified cluster sampling approach was used to collect medical record data of patients from 595 hospitals (26 regional, 70 provincial, 499 community) from all 77 provinces in Thailand between April 1 to June 30, 2012 (www.damus.in.th). Our study is part of an ongoing project called “An assessment on quality of care among patients diagnosed with type 2 diabetes and hypertension visiting hospitals of Ministry of Public Health and Bangkok Metropolitan Administration in Thailand”. Overall, 26,860 patients, aged 35 years or older, with type 2 diabetes mellitus (T2DM) were identified who had at least one visit at either a specialist diabetes clinic (SDC, *n* = 20,185) or a general medical outpatient clinic (GMC, *n* = 6675). Clinical information from patients’ medical records were routinely collected by hospital staff, which were then transcribed to case report forms by clinical research associates. Permission to use these data was obtained from the Thailand Medical Research Network (MedResNet) in 2012 and approval to conduct our research was obtained from the Ethics Committee for Human Research of Khon Kaen University, Thailand.

For the present study, the outcome variables of interest were split into two main groups: Clinical targets and processes of care. Clinical targets include the ABC of diabetes, A-glycated haemoglobin (HbA1c), B-blood pressure (BP), and C-low density lipoprotein cholesterol (LDL-C). Processes of diabetes care include the FACE of diabetes, where F represents Foot exam, A-glycated haemoglobin (HbA1c) exam, C-low density lipoprotein cholesterol (LDL-C) exam, and E-eye exam. In addition, aggregates of the quality of care measures were generated for clinical targets (*AllABC)*, processes of care (*AllFACE)*, and all quality of care indicators (a combination of process of care-FACE of diabetes and clinical target-ABC of diabetes) *All7Q* and *Num7Q*. *AllABC* represents all three of the treatment goals achieved (yes/no). *AllFACE* represents an indicator variable for cases where *all four* processes of care were conducted (yes/no). *All7Q* represents an indicator variable of whether all seven of the clinical indicators were achieved (yes/no), and finally the count variable, *Num7Q* represents the number of clinical outcome and process of care examinations achieved *(0, 1, 2, 3, 4, 5, 6, or 7).* Patients were considered satisfactory for achievement of clinical targets if A-glycated haemoglobin (HbA1c) < 7.0% (53 mol/mmol) (yes/no) [11], B-blood pressure (BP) <140/80 mmHg (yes/no), and C-low density lipoprotein cholesterol (LDL-C) <100 mg/dL (yes/no) [12]. Patients were considered to satisfactorily meet the examination target if they were examined twice in the least 12 months for HbA1c (yes/no), and once in the previous 12 months for cholesterol (yes/no), foot (yes/no) and eyes (yes/no) [12].

In this study, the main effect of interest was the clinic types patients attended specialized diabetes clinic-SDC, or general medical clinic-GMC. Other variables we considered as potentially important predictors or confounders included: Age, sex (female and male), medical coverage type (universal coverage, civil security medical benefit (CSBM), social security scheme, and other), religion (Buddism or Muslim), Body Mass Index (BMI) class, duration of type 2 diabetes mellitus (both measured continuously), hypertension, and Hospital type (Regional, Provincial, Community).

### Statistical analysis

Means and standard deviations were used to summarize continuous patient characteristics and frequencies and percentages were employed for those that categorical. As the data employed in our study are multilevel, with some covariates measured at the hospital level, and others at the patient level. To account for this multi-level structure, mixed effect modeling was employed, a model also able to account for any hospital clustering effect.

The associations of three aggregates (*All ABC*, *All FACE*, *All7Q*) and seven individual measures (clinical outcomes is A, B, C, and processes of care is F, A, C, E) with clinic type were investigated by using Binary Logistic Mixed Effect Regression. The count outcome, *Num7Q,* was modeled using a Poisson Mixed Effect Regression.

Multivariable models were selected using the purposeful selection of covariates (PSC) [13] approach, we used this approach as PSC, unlike many other algothimic model building procedures can identify and control for confounders. Most covariates were included (or excluded) in the final model based on whether they were either independent risk factors, or confounders. Clinic type (SDC or GMC) and Hospital type (Community, Provincial, Regional), however, were the study effects and were consequently forced into all models. We also investigated whether hospital type modified the effect of clinic type by testing for interactions between these two predictors. To probe significant clinic type-hospital type interactions, a subsequent subgroup analyses were performed. All data analysis was conducted using R version 3.1.2 [14] and the R library lme4 (1.1–7) [15] was used to perform the mixed effect modelling.

## Results

Overall, the study sample consisted of 26,860 type 2 diabetes mellitus patients, of which 75% of the patients attended specialized diabetes clinics-SDCs and 25% of the patients attended general medical clinics-GMCs. In regional hospitals approximately half of the T2DM patients attended specialist clinics (47%), whereas this proportion increased considerably in provincial and community hospitals (64% and 88%, respectively).

Patients’ characteristics for each hospital types are provided in Table 1. Both overall (hospital types combined), and for the individual levels of hospitals (regional, provincial, and community), patients attending the SDCs tended to be higher in the other insurance category [out-of-pocket], whereas patients attending the GMCs had a higher prevalence of hypertension and obesity (BMI > =30 kg/m^3^).

**Table 1:** Patient characteristics in specialist diabetes clinics (SDCs) and general medical clinics (GMCs) at hospital levels

	Overall		Regional		Provincial		Community	
			*n* = 3569		*n* = 5517		*n* = 16,384	
	SDCs	GMCs	SDCs	GMCs	SDCs	GMCs	SDCs	GMCs
	*N* = 20,185^a^	N= 6675^a^	N= 1678^a^	N= 1891^a^	N= 3518^a^	N= 1999^a^	*N* = 14,453^a^	N=1931^a^
	(75%)	(25%)	(47.02)	(52.98)	(63.77)	(36.23)	(88.21)	(11.79)
Female n (%)	14,285 (70.8)	4554 (68.3)	1118 (66.6)	1317 (69.7)	2423 (68.9)	1332 (66.7)	10,390 (71.9)	1354 (70.1)
Age [years] Mean (SD)	59.58 (10.7)	60.42 (10.9)	60.5 (10.6)	61.2 (11.1)	60.4 (10.66)	60.6 (10.95)	59.3 (10.7)	59.6 (10.3)
Duration [years] Mean(SD)	7.64 (4.6)	7.24 (4.6)	9.1 (5.8)	7.4 (4.9)	8.1 (4.62)	7.1 (4.28)	7.4 (4.5)	7.6 (4.6)
Hypertension n (%)	13,250 (65.6)	4957 (74.3)	1238 (73.8)	1522 (80.5)	2481 (70.5)	1447 (72.4)	9162 (63.4)	1321 (68.4)
Religion n (%)								
Buddhism	17,734 (96.1)	5729 (97.3)	1468 (97.4)	1539 (98.3)	2945 (95.1)	1620 (97.5)	12,927 (96.4)	1856 (99.4)
Muslim	723 (3.9)	161 (2.7)	39 (2.6)	26 (1.7)	152 (4.9)	41 (2.5)	480 (3.6)	12 (0.6)
Scheme n (%)								
Universal coverage	11,416 (57)	3935 (59.9)	960 (58.3)	1162 (62.1)	1931 (55.3)	1072 (54.4)	8057 (56.1)	1078 (56.0)
CSMB	3045 (15.2)	1370 (20.8)	533 (32.3)	480 (25.6)	833 (23.9)	561 (28.5)	1666 (11.6)	254 (13.2)
Social security	522 (2.6)	383 (5.8)	62 (3.76)	126 (6.7)	143 (4.1)	108 (5.5)	273 (1.9)	41 (2.1)
Other[out of pocket]	5046 (25.2)	887 (13.5)	93 (5.6)	104 (5.5)	585 (16.7)	228 (11.6)	4367 (30.4)	552 (28.7)
BMI (kg/m^2^) n (%)								
< 18.50	712 (3.7)	167 (2.9)	53 (3.5)	39 (2.8)	118 (3.5)	46 (2.6)	529 (3.8)	60 (3.3)
18.5–22.9	5116 (26.4)	1326 (23.3)	361 (23.6)	305 (21.9)	785 (23.4)	414 (23.2)	3879 (27.7)	469 (25.9)
23.0–24.9	3980 (20.6)	1156 (20.3)	320 (20.9)	252 (18.1)	660 (19.7)	382 (21.4)	2907 (20.8)	387 (21.3)
25.0–29.9	7059 (36.5)	2101 (36.9)	575 (37.6)	557 (39.9)	1300 (38.7)	631 (35.4)	4992 (35.7)	645 (35.6)
> = 30.0	2488 (12.9)	938 (16.5)	221 (14.4)	242 (17.3)	494 (14.7)	309 (17.3)	1686 (12.0)	252 (13.9)

*Abbreviations*: *n* number, % percentage, kg/m^2^ kilograms per meter squared, *SD* standard deviation, *GMCs* general medical clinics, *SDCs* specialist diabetes clinics, *CSMB* civil servant medical benefit, *BMI* Body Mass Index

^a^Values used as denominator of prevalence calculations

For variables where patient information is missing, the denominator is adjusted accordingly

The relative performance of processes of care and clinical targets are illustrated in Table 2. In our sample, achievement of both *All FACE* and *All7Q* were higher in SDCs compared to GMCs, regardless of hospital type. In contrast the reverse seemed to be the case for *All ABC*, where GMCs appeared to perform better for community and provincial hospitals. However, in regional hospitals SDCs seemed to outperform GMCs for the achievement of all three clinical targets.

**Table 2:** Achievement of quality of care among type 2 diabetes in SDCs and GMCs at hospital levels

	Overall		Regional hospital		Provincial hospital		Community hospital	
Quality of care	SDCs	GMCs	SDCs	GMCs	SDCs	GMCs	SDCs	GMCs
	N^a^ (%)	N^a^ (%)	N^a^ (%)	N^a^ (%)	N^a^ (%)	N^a^ (%)	N^a^ (%)	N^a^ (%)
HbA1c exam	1,5061 (74.6)	4908 (73.5)	1614 (96.2)	1588 (83.9)	2945 (83.7)	1417 (70.9)	10,217 (70.7)	1276 (66.1)
LDL-C exam	17,248 (85.5)	5283 (79.2)	1577 (93.9)	1608 (85.0)	3068 (87.2)	1493 (74.7)	12,298 (85.1)	1502 (77.8)
Eye exam	10,494 (52.5)	2821 (43.1)	1067 (63.9)	900 (48.5)	2060 (58.9)	826 (42.4)	7190 (50.3)	761 (40.1)
Foot exam	13,655 (68.3)	3076 (46.9)	1067 (63.9)	861 (46.4)	2307 (66.1)	649 (33.3)	9996 (69.9)	1074 (56.5)
AllFACE	6437 (32.2)	1245 (19.0)	800 (47.9)	476 (25.6)	1361 (38.9)	224 (11.5)	4229 (29.6)	366 (19.3)
HbA1c <7%(53 mmol/mol)	5016 (33.83)	1760 (36.92)	562 (35.1)	573 (36.9)	974 (33.4)	519 (38.2)	3370 (33.5)	416 (33.4)
BP < 140/80 mmHg	10,584 (53.04)	3178 (48.57)	961 (57.6)	875 (47.2)	1775 (50.9)	873 (44.8)	7647 (53.6)	1060 (55.8)
LDL-C < 100 mg/dL	7024 (41.80)	2351 (46.14)	794 (50.9)	772 (49.4)	1411 (46.9)	681 (47.7)	4707 (39.4)	608 (42.1)
AllABC	1244 (9.09)	401 (9.82)	191 (12.6)	124 (8.89)	262 (9.7)	105 (9.6)	776 (8.4)	113 (10.5)
Number of 7Q								
2	458 (3.35)	239 (5.86)	55 (3.6)	84 (6.0)	89 (3.3)	75 (6.9)	308 (3.3)	58 (5.4)
3	1971 (14.41)	759 (18.60)	190 (12.5)	261 (18.7)	344 (12.8)	238 (21.8)	1390 (15.0)	175 (16.2)
4	3863 (28.24)	1256 (30.78)	355 (23.4)	401 (28.8)	748 (27.8)	369 (33.9)	2679 (28.9)	331 (30.7)
5	4338 (31.71)	1149 (28.16)	494 (32.5)	398 (28.5)	846 (31.5)	279 (25.6)	1585 (31.7)	316 (29.3)
6	2443 (17.86)	540 (13.24)	315 (20.7)	190 (13.6)	523 (19.4)	102 (9.4)	356 (17.1)	166 (15.4)
All7Q	608 (4.44)	137 (3.36)	111 (7.3)	60 (4.3)	139 (5.2)	26 (2.4)	356 (3.8)	31 (2.9)

*Abbreviations*: *n* number, % percentage; *CI* confident interval, *HbA1c* glycated hemoglobin, *BP* blood pressure, *LDL-C* low-density lipoprotein cholesterol, ABC A-HbA1c, B- BP, C-LDL-C, *GMCs* general medical clinics, *SDCs* specialist diabetes clinics

^a^Percentages are based on available case analysis

Where a patient had a missing value they were excluded from both the numerator and the denominator of the prevalence calculation

Table 3 provides the case-mix adjusted model for all nine indicators, and Table 4 provides both the crude and case-mix adjusted models for clinic type. Table 4 also provides the subgroup analysis which examines the clinic type effect for the different health care setting (regional, provincial and community hospitals). Although the crude effect of clinic type was identified as significant for LDL-C, eye exam and *All7Q*, the clinic type effect did not retain significance after adjusting for case-mix. The significance of clinic effect for all other indicators remained the same after adjustment for case-mix, whether originally significant, or non-significant.

**Table 3:** Multivariate multilevel logistic regression for a combination of processes of care and clinical targets

Factor	Processes of care					Clinical targets				A combination	
	HbA1c exam	LDL-C exam	Eye exam	Foot exam	AllFACE	HbA1c	BP	LDL-C	AllABC	All7Q	Num7Q
	AOR (95% CI)	AOR (95% CI)	AOR (95% CI)	AOR (95% CI)	AOR (95% CI)	AOR (95% CI)	AOR (95% CI)	AOR (95% CI)	AOR (95% CI)	AOR (95% CI)	ARR (95% CI)
Specialist clinics	3.60 (2.23–5.80)	0.97 (0.63–1.51)	1.14 (0.88–1.48)	1.87 (1.40–2.51)	1.89 (1.42–2.53)	0.94 (0.72–1.23)	1.41 (1.12–1.76)	1.24 (0.98–1.58)	1.25 (0.86–1.82)	1.22 (0.74–2.00)	1.06 (1.01–1.11)
Female	-	-	1.20 (1.12–1.29)	1.12 (1.04–1.21)	1.11 (1.03–1.21)	0.88 (0.82–0.96)	1.21 (1.14–1.29)	0.74 (0.69–0.79)	0.91 (0.80–1.03)	-	-
Age [10 years]	-	0.96 (0.91–1.02)	-	-	-	1.42 (1.36–1.48)	1.03 (1.01–1.06)	1.04 (1.01–1.08)	1.32 (1.23–1.41)	1.31 (1.21–1.42)	1.02 (1.01–1.03)
Buddhism	-	1.08 (0.77–1.52)	-	-	1.15 (0.90–1.48)	1.17 (0.93–1.49)	-	1.40 (1.14–1.73)	1.30 (0.86–1.96)	-	-
Duration [5 years]	-	0.98 (0.93–1.04)	1.09 (1.05–1.13)	1.10 (1.05–1.14)	1.06 (1.02–1.11)	0.68 (0.65–0.71)	1.06 (1.03–1.09)	1.03 (0.99–1.07)	0.84 (0.78–0.90)	0.87 (0.79–0.96)	-
Hypertension	-	1.17 (1.05–1.32)	-		0.68 (0.33–1.02)	1.25 (1.15–1.36)	0.50 (0.47–0.53)	1.09 (1.01–1.18)	0.90 (0.78–1.03)	-	-
Scheme	*p* < 0.001	*p* < 0.001	*p* < 0.001	*p* < 0.001	*p* < 0.001	*p* < 0.001	-	*p* < 0.001	*p* < 0.001	-	-
Civil servant	1.11 (0.99–1.25)	1.11 (0.96–1.28)	0.85 (0.78–0.94)	0.76 (0.69–0.84)	0.86 (0.77–0.95)	1.23 (1.11–1.36)	-	1.08 (0.98–1.18)	1.20 (1.02–1.41)	-	-
Social security	1.01 (0.80–1.28)	0.77 (0.58–1.04)	0.88 (0.73–1.07)	0.81 (0.66–1.01)	0.78 (0.61–0.98)	1.08 (0.86–1.35)	-	0.93 (0.77–1.13)	1.01 (0.69–1.48)	-	-
Other	1.07 (0.97–1.18)	1.03 (0.89–1.18)	1.11 (1.01–1.21)	1.04 (0.94–1.15)	1.09 (0.99–1.21)	1.03 (0.93–1.14)	-	1.00 (0.91–1.09)	1.05 (0.89–1.24)	-	-
BMI [kg/m2]	*p* < 0.001	*p* < 0.001	*p* < 0.001	*p* < 0.001	*p* < 0.001	*p* < 0.001	*p* < 0.001	*p* < 0.001	*p* < 0.001	*p* < 0.001	*p* < 0.001
18.5–22.9	0.97 (0.79–1.20)	1.01 (0.77–1.31)	1.26 (1.04–1.51)	1.15 (0.95–1.40)	1.20 (0.97–1.48)	1.00 (0.82–1.23)	0.85 (0.72–1.00)	0.86 (0.72–1.03)	1.01 (0.75–1.37)	1.10 (0.72–1.68)	0.99 (0.95–1.03)
23.0–24.9	1.12 (0.91–1.39)	1.16 (0.89–1.52)	1.26 (1.05–1.52)	1.22 (1.00–1.49)	1.27 (1.02–1.58)	0.90 (0.74–1.12)	0.67 (0.56–0.79)	0.80 (0.67–0.96)	0.82 (0.60–1.11)	0.85 (0.55–1.31)	0.97 (0.93–1.01)
25.0–29.9	1.08 (0.88–1.32)	1.09 (0.84–1.41)	1.26 (1.05–1.51)	1.12 (0.92–1.36)	1.23 (1.00–1.52)	0.80 (0.66–0.99)	0.55 (0.47–0.65)	0.79 (0.67–0.95)	0.74 (0.54–0.99)	0.79 (0.52–1.20)	0.96 (0.92–1.00)
> = 30.0	1.10 (0.88–1.38)	1.22 (0.91–1.62)	1.35 (1.11–1.64)	1.27 (1.03–1.57)	1.45 (1.16–1.81)	0.82 (0.67–1.03)	0.42 (0.35–0.50)	0.75 (0.62–0.90)	0.60 (0.42–0.85)	0.66 (0.41–1.06)	0.94 (0.90–0.99)
Hospital type	*p* < 0.001	*p* < 0.001	*p* < 0.001	*p* < 0.001	*p* < 0.001	*p* < 0.001	*p* < 0.001	*p* < 0.001	*p* < 0.001	*p* < 0.001	*p* < 0.001
Provincial	0.36 (0.12–1.03)	0.52 (0.20–1.37)	0.50 (0.19–1.32)	0.40 (0.13–1.21)	0.15 (0.05–0.43)	0.95 (0.62–1.48)	0.72 (0.50–1.03)	0.81 (0.57–1.14)	0.85 (0.50–1.42)	0.39 (0.17–0.88)	0.93 (0.86–0.99)
Community	0.34 (0.13–0.87)	0.57 (0.24–1.37)	0.25 (0.11–0.60)	1.24 (0.47–3.32)	0.20 (0.08–0.50)	0.84 (0.56–1.26)	1.29 (0.93–1.79)	0.77 (0.56–1.05)	1.02 (0.64–1.64)	0.43 (0.20–0.91)	0.98 (0.92–1.05)
Hospital^a^Clinics	*p* < 0.001	*p* = 0.36	*p* < 0.001	*p* < 0.001	*p* < 0.001	*p* = 0.8	*p* < 0.001	*p* < 0.05	*p* = 0.07	*p* = 0.36	*p* = 0.09
SDCs: Provincial	0.59 (0.34–1.03)	1.00 (0.56–1.76)	1.83 (1.23–2.73)	2.72 (1.73–4.27)	2.83 (1.66–4.81)	0.99 (0.67–1.46)	1.10 (0.81–1.50)	1.03 (0.73–1.45)	0.88 (0.51–1.51)	1.68 (0.75–3.72)	1.05 (0.98–1.13)
SDCs: Community	0.26 (0.15–0.47)	1.38 (0.79–2.43)	2.20 (1.45–3.33)	1.19 (0.76–1.87)	1.28 (0.78–2.11)	1.09 (0.76–1.56)	0.58 (0.44–0.78)	0.70 (0.52–0.95)	0.59 (0.36–0.95)	1.00 (0.48–2.08)	0.97 (0.91–1.04)

^a^General medical clinics are as reference category

Abbreviations: *n* number, *AOR* adjusted odd ratio, *ARR* adjusted relative risk, *CI* confident interval, *HbA1c* glycated hemoglobin, *BP* blood pressure, *LDL-C* low-density lipoprotein cholesterol, *ABC* A-HbA1c, B- BP, C-LDL-C, *GMCs* general medical clinics, *SDCs* specialist diabetes clinics, *CSMB* civil servant medical benefit, *BMI*, body mass index

**Table 4:** Mixed effect logistic regression for a combination of processes of care and clinical targets on differences between SDCs and GMCs^a^ “clinics effect” at each hospital level and differences between clinic type effects across hospital levels (clinics-hospital interaction effect)

	Case-mix		Regional		Provincial		Community	
	Unadjusted	Adjusted	Unadjusted	Adjusted	Unadjusted	Adjusted	Unadjusted	Adjusted
	OR (95% CI)	OR (95% CI)	OR (95% CI)	OR (95% CI)	OR (95% CI)	OR (95% CI)	OR (95% CI)	OR (95% CI)
Processes of care								
HbA1c exam	1.96 (1.64–2.34)	3.60 (2.23–5.80)	4.17 (2.83–6.13)	3.24 (2.08–5.03)	2.57 (1.97–3.36)	2.55 (1.88–3.46)	0.97 (0.60–1.36)	0.98 (0.69–1.41)
LDL-C exam	1.26 (1.05–1.51)	0.97 (0.63–1.51)	1.47 (1.06–2.05)	1.13 (0.74–1.72)	1.20 (0.89–1.62)	0.89 (0.63–1.25)	1.39 (1.01–1.94)	1.33 (0.93–1.89)
Eye exam	1.87 (1.62–2.17)	1.14 (0.88–1.48)	1.49 (1.20–1.85)	1.35 (1.04–1.77)	2.35 (1.80–3.06)	1.99 (1.48–2.69)	2.29 (1.67–3.15)	2.54 (1.82–3.55)
Foot exam	3.37 (2.85–3.97)	1.87 (1.40–2.51)	2.97 (2.31–3.83)	2.37 (1.73–3.25)	5.74 (4.21–7.82)	7.01 (4.99–9.84)	2.21 (1.59–3.06)	2.25 (1.62–3.13)
AllFACE	2.73 (2.29–3.26)	1.89 (1.42–2.53)	2.42 (1.88–3.13)	1.68 (1.26–2.24)	2.11 (1.54–3.12)	2.14 (1.50–3.06)	1.46 (0.92–2.33)	1.50 (0.91–2.45)
Clinical outcome								
HbA1c < 7% (53 mmol/mol)	0.90 (0.80–1.02)	0.94 (0.72–1.23)	0.90 (0.73–1.11)	0.89 (0.69–1.15)	0.89 (0.71–1.12)	0.92 (0.70–1.19)	1.00 (0.79–1.28)	1.02 (0.79–1.31)
BP < 140/80 mmHg	1.24 (1.12–1.37)	1.41 (1.12–1.76)	1.42 (1.17–1.71)	1.55 (1.25–1.92)	1.57 (1.29–1.91)	1.52 (1.23–1.88)	0.92 (0.78–1.09)	0.81 (0.67–0.98)
LDL-C < 100 mg/dL	1.05 (0.94–1.17)	1.24 (0.98–1.58)	1.30 (1.07–1.59)	1.19 (0.96–1.47)	1.27 (1.02–1.57)	1.28 (1.03–1.59)	0.84 (0.70–1.01)	0.87 (0.72–1.05)
AllABC	0.93 (0.79–1.12)	1.25 (0.86–1.82)	1.37 (0.99–1.87)	1.28 (0.90–1.84)	1.05 (0.75–1.46)	1.06 (0.74–1.52)	0.77 (0.57–1.05)	0.72 (0.52–1.01)
A combination								
All7Q	1.33 (1.01–1.78)	1.22 (0.74–2.00)	1.43 (0.91–2.24)	1.23 (0.76–1.99)	2.01 (1.12–3.63)	1.91 (1.06–3.46)	1.26 (0.74–2.14)	1.26 (0.72–2.20)
Num7Q^b^	1.06 (1.04–1.09)	1.06 (1.01–1.11)	1.09 (1.03–1.14)	1.06 (1.00–1.11)	1.12 (1.05–1.18)	1.11 (1.05–1.18)	1.03 (0.99–1.07)	1.03 (0.99–1.08)

^a^General medical clinics are as reference category

^b^Num7Q count is relative risk

*Abbreviations*: *n* number, *AOR* Adjusted odd ratio, *ARR* adjusted relative risk, *CI* confident interval, *HbA1c* glycated hemoglobin, *BP* blood pressure, *LDL-C* low-density lipoprotein cholesterol, *ABC* A-HbA1c, B- BP, C-LDL-C, *GMCs* general medical clinics, *SDCs* specialist diabetes clinics

In multivariate binary logistic mixed effect regression analyses (Table 3), we found that case-mix adjustment had little impact on the efficacy of the type of clinic attended with unadjusted and adjusted odds ratios being similar. Overall, perusal of the patient level effects for the multivariate model revealed a strong consistency in the direction of association for processes of care, clinical targets, and their combination. Females tended to achieve the eye exam, foot exam, *All FACE*, and blood pressure < 140/80 mmHg clinical targets more often, but were less likely to achieve the HbA1c <7% (53 mmol/mol) and LDL-C < 100 mg/dL clinical targets. Age was associated with all clinical targets with older patients more likely to achieve HbA1c <7% (53 mmol/mol), BP <140/80 mmHg, LDL-C < 100 mg/dL, *All ABC.* Older age was also indicated as protective in terms of the quality of care aggregates, *All7Q*, and *Num7Q*. Higher diabetes duration was associated with a lower likelihood of achieving the HbA1c <7% (53 mmol/mol), *All ABC* and *All7Q*, but positively associated with achieving eye exam, foot exam, *All FACE*, and BP < 140/80 mmHg. Hypertensive patients were more likely to achieve the LDL-C exam, HbA1c <7% (53 mmol/mol) and LDL-C < 100 mg/dL but, perhaps not surprisingly, were much less likely to achieve the BP < 140/80 mmHg. Typically, BMI was higher in patients who had eye exam and *AllFACE* exam, but BMI was negatively association with achievement of the BP < 140/80 mmHg and LDL-C < 100 mg/dL.

When patients with T2DM from the different hospital levels (community, provincial and regional) are pooled, there is evidence that type of clinic had an effect on the achievement of several of the processes of care, the clinical targets, and a combination of processes of care and clinical targets (Table 3). Perusal of the Table 4 however, shows the nature of differences between general medical clinics and specialist diabetes clinics varies with the type of hospital (community, provincial and regional) for HbA1c exam, eye exam, foot exam, *All FACE* exam, BP < 140/80 mmHg and LDL-C < 100 mg/dL. In contrast, there is no evidence to suggest that the clinic types effect varied with hospital type for LDL-C exam, HbA1c target <7% (53 mmol/mol), *AllABC*, *All7Q*, and *Num7Q*.

The sub-group analysis (Table 4) reveals that regardless of the type of hospital T2DM patients attend (regional, provincial, community)-SDCs were considerably more successful in achieving of eye and foot exams. Type 2 diabetes mellitus patients attending larger hospital (regional and provincial)-SDCs were more likely to achieve both HbA1c exam and *All FACE* exam (AOR regional = 3.24, 95% CI [2.08, 5.03]; AOR provincial = 2.55, 95% CI [1.87, 3.46]), the BP clinical target <140/80 mmHg (AOR regional = 1.55, 95% CI [1.25, 1.92]; AOR provincial = 1.52, 95% CI [1.23, 1.88]), and to achieve higher *Num7Q* than patients attending larger hospital-based general clinics (ARR regional = 1.06, 95% CI [1.00,1.11]; ARR provincial = 1.11, 95% CI [1.05,1.18]). However, this trend was reversed for achievement of BP <140/80 mmHg where achievement was lower in specialist diabetes clinics (AOR community = 0.81, 95% CI [0.67, 0.98]) (Table 4). This pattern was also evident for the cholesterol clinical target <100 mg/dL and *All7Q*, although the odds of achieving LDL-C and *All7Q* in specialized clinics was not statistically higher in regional hospitals, or statistically lower in community hospitals. That is, in terms of the LDL-C clinical target and *All7Q*, specialized clinics can only be shown to differ in provincial hospitals (AOR provincial =1.28, 95% CI [1.03, 1.59], and AOR provincial =1.91, 95% CI [1.06, 3.46], respectively) (Table 4).

## Discussion

We compare the performance of general medical clinics (GMCs) and specialist diabetes clinics (SDCs) at different types of hospitals, for quality of diabetes care. Preliminary investigation suggested that SDCs outperform GMCs in achieving various processes of care (HbA1c exam, foot exam, and All FACE), clinical target (blood pressure target), and combination of process of care and clinical target (*the Num7Q*). However, we found that hospital type modified the relative efficacy of specialist clinics and only when a subsequent subgroup analysis by hospital type (regional, provincial, and community) was performed did the true nature of difference between GMCs and SDCs become apparent. For process of care examination, we found that patients who attended SDCs were had a substantially higher chance of achieving of eye and foot exams, regardless of hospital type. However, beyond this, the magnitude, and even direction, of differences between SDCs and GMCs varied with hospital type. Larger hospital (regional and provincial)-SDCs were associated with higher levels of achievement of HbA1c exam and *AllFACE*. In terms of the achievement of clinical targets, larger hospital (regional and provincial)-SDCs were associated with better achievement of the BP target. Interestingly, this trend was the same for LDL-C target but unlike BP, LDL-C was could only be demonstrated a statistically significantly different at provincial hospitals. Interestingly, we found that reverse is true at community hospitals, where the achievement of BP clinical target was better at GMCs.

The performance of clinics has been shown to be associated with the quality of diabetes care. Previous studies have showed that the specialist diabetes clinics generally outperform general medical clinics in quality of diabetes care [10, 16]. This present study adds new information to help explain the exact nature of differences in quality of diabetes care between patients attending specialist diabetes clinics and those attending general medical clinics at different hospital types (regional, provincial, and community). This study demonstrates that larger hospital (regional and provincial) specialist diabetes clinics outperform general medical clinics in terms of the number of quality of diabetes care indicators achieved (Num7Q). However, in terms of achievement of all seven indicators (three clinical targets and four processes of care), we found that only the medium sized provincial hospitals’ specialized diabetes clinics could be shown to be superior to general medical clinics.

There are a number of potential reasons why large hospital-SDCs generally outperformed larger hospital-GMCs in term of quality of diabetes care. First, this difference may be due to the composition of SDCs teams which include specialists [17]. Second, SDC physicians are more likely to be familiar with, or better adhere to the standard practice guidelines, relative to GMC physicians [18]. Third, patients with T2DM cared for by SDCs may also have better access to the exam facilities and other infrastructure available in larger hospitals. Interestingly, we could not demonstrate that the smaller community level hospitals SDCs were superior to community hospital GMCs. A possible explanation for this that while small hospitals SDCs might be dedicated solely to the care of diabetes patients, these hospitals are less likely to have the teams of specialists that we would normally associate with patient care of advanced type 2 diabetes patients, such as those that have developed serious type 2 diabetes complications. Instead, patients with more advanced chronic complications would be more likely to be refereed to larger hospitals. To the best of our knowledge, no other study has considered aggregated measures of diabetes patient quality of care that include both clinical target and process of care components. Our study is the first to consider such indicators holistically in terms of identifying strategies of optimal care and T2DM patients care at a reasonable cost.

Unfortunately, we had no patient or hospital cost data to gauge the cost-benefit of the higher efficacy of large hospitals specialized diabetes clinics against the added costs of this more advanced form of patients care. We feel this is an important avenue that needs further investigation.

Aggregate measures are perhaps the most important when health insurer, health care organizations or providers teams need to evaluate the performance of hospital-clinics both in terms of processes of care provided, and clinical outcome achieved. Furthermore, different organization or stakeholders may have different opinions or information needs [19]. In particular, our study provides strong justification for the use of clinical target and process of care aggregate information useful for stakeholder or organizations in terms of quality diabetes care achieved, and a useful tool in how to investigate the efficacy of interventions or impacts of policy decisions in terms of patients care and outcomes. Although, we did not investigate the associated costs with particular structure of care approaches, our study has shown that clinical target-process aggregate measures we used differ across different structures of care, and these can now be measured in terms of patient and hospital level costs.

Our study did have some potential limitations. First, our study was cross-sectional, so measures of clinical targets, processes of care and patient characteristics were measure concurrently. We had no information about the history of patient clinic type attendance (for example, they may have just changed to their current type of clinic). Second, It is likely that process of care and clinical target performed on admission, and clinic type and hospital level attended are based on type of medical coverage (universal coverage, CSMB, social security scheme, and out of pocket) suggesting that medical coverage type, which is largely driven social-economically, may potentially confound both the hospital and the clinic type effect, although, our modeling approach did attempt to statistically adjust for the coverage type effect, along with other important socio-economic factors. Third, our retrospective study design did not include important lifestyle variables like exercise and diet. Furthermore, our multivariate modeling was based on complete-case analysis and consequently some information bias may have been introduced if data were not missing at random.

Finally, although we have a strong study design in terms of population coverage with an appropriate sampling design used to capture a representative cross-section of Thai Type 2 diabetes outpatients, the generalizability of our results to health care settings in other middle income countries, within and outside Southeast Asia is unknown. Furthermore, whether our findings are relevant to more resource-rich or resource-poor setting is unknown. With so few studies conducted that combine clinical target and process of care indicators, it is difficult to gauge whether our findings are likely to be valid outside Thailand.

The present study also had some strengths. First, our data were obtained from a large and nationally representative sample of T2DM patients attending 595 hospitals across Thailand. Second, we used a strong methodological approach in our analysis which controlled for the hospital clustering effect, a study design artifact that few studies in this area consider. In addition, few multi-center studies that consider type 2 diabetes mellitus patients quality of care account for the multilevel nature of their data (patients within hospitals, hospitals within hospital types). Finally, we showed the use of mixed models for the analysis of these types of clustered and multilevel data.

## Conclusions

SDCs cannot be said to be universally better than GMCs in terms of patient quality of care. Indeed, we found that the nature of differences between SDCs and GMCs varied with hospital type. In general, larger hospital SDCs perform better than larger hospital GMCs. At smaller hospitals, there was either no difference (most indicators) or GMCs were actually superior (BP target). Further study is needed is to identify which aspects of general medical and specialist diabetes clinics lead to a superior model of care delivery or to reduce mortality and health cost or further research. Furthermore, research needs to be done in other countries with either similar or different levels of resourcing for their health care sector, to examine whether our findings hold across different health care settings.

## Abbreviations

ABC: A-HbA1c, B- BP, C-LDL-C
BMI: Body mass index
BP: Blood pressure
CRFs: Case record forms
CSMB: Civil servant medical benefit
DMHT: Diabetes mellitus and Hypertension
GLMM: Generalized linear mixed modeling
GMCs: General medical clinics
HbA1c: Glycated haemoglobin
LDL-C: Low-density lipoprotein cholesterol
MedResNet: Medical Research Network
PSC: Purposeful selection of covariates
SDCs: Specialist diabetes clinics
T2DM: Type 2 diabetes mellitus
The FACE: F-Foot exam, A-Glycated haemoglobin (HbA1c) exam, C-Low density lipoprotein cholesterol (LDL-C) exam, and E-Eye exam
